# Largescale population genomics versus deep phenotyping: Brute force or elegant pragmatism towards precision medicine

**DOI:** 10.1038/s41525-019-0080-0

**Published:** 2019-03-26

**Authors:** Lamis Yehia, Charis Eng

**Affiliations:** 1 Genomic Medicine Institute, Lerner Research Institute, Cleveland Clinic, Cleveland, OH 44195 USA; 2 Taussig Cancer Institute, Cleveland Clinic, Cleveland, OH 44195 USA; 3 Department of Genetics and Genome Sciences, Case Western Reserve University School of Medicine, Cleveland, OH 44106 USA; 4 Germline High Risk Cancer Focus Group, CASE Comprehensive Cancer Center, Case Western Reserve University, Cleveland, OH 44106 USA

Whenever you find yourself on the side of the majority, it is time to pause and reflect.

– Mark Twain

Biomedical research has been accelerating at an unprecedented pace, with evidence racing towards advancing precision medicine initiatives worldwide.^1^ Genomics is at the center stage of these efforts, with extensive genetic and genomic data being continuously collected, analysed, and archived. Indeed, over the past decade, we have deepened our understanding of the underlying genetic etiologies and biologic mechanisms of both, rare Mendelian and common complex human diseases. One critical issue, rightly identified by many, is the value and translational utility of the massive research-associated -omics data, particularly as related to identifying robust genotype-phenotype associations that could impact patient care and outcomes. In other words, have we blinded ourselves with big conglomerated data that we cannot see the trees for the forest?

Large-scale population-based studies and associated consortia have played a pivotal role in laying the genomic framework of various diseased and healthy populations. Resultant data include both common and rare variants associated with different phenotypic states. The genome-wide association studies (GWAS) approach has emerged as a powerful tool for identifying genomic loci for various common human diseases and traits. Since its inception in 2008, the GWAS Catalog now includes >100,000 SNP-trait associations.^2^ And while there is great success in mapping putative common risk alleles, more research is required to pinpoint the genes involved. Relatedly, the surge in next-generation sequencing capabilities combined with a decline in sequencing cost and optimized computational infrastructure have made it practical to sequence humans at the population level. Such efforts have also led to the establishment of population level, publicly accessible databases to facilitate data sharing and discovery. A recent example has been presented through the Exome Aggregation Consortium (ExAC), followed by The Genome Aggregation Database (gnomAD), the largest public catalogue of 141,456 individuals sequenced as part of various disease-related or population genetic studies.^3^ Such large-scale genomic datasets of diverse human populations indeed form a critical framework for the functional interpretation of genetic variations, both in the research and clinical settings.^4^ Although such a gestalt approach provides a powerful tool to hone in on disease-causing variations, including ultra-rare ones, the utility of this and other population databases is naturally context-dependent. As such, germline disease-associated variants in *TP53* have been found to be enriched in ExAC and gnomAD populations.^5^ Other pathogenic variants were found in known hereditary cancer predisposition genes such as *PTEN*, *BRCA1*, *BRCA2*, *APC* and *MLH1*. Based on overall allele number in the interrogated populations, these rare disease-causing variants would still hypothetically represent a lower overall burden compared to a purely diseased population. However, the counterargument is the fact that such population databases do include individuals with (e.g. TCGA) or projected to have cancer, and these individuals may indeed be undiagnosed cases harboring *bona fide*, yet unsuspected, germline high penetrance mutations. Understandably, the power harnessed from an ever-increasing sample size is countered by an inability to obtain individual-level genotypic or phenotypic data to tease out such associations. This is particularly important in the context of more common phenotypes such as cancer and heart disease, although efforts have been made to stratify population genetic data by global phenotypic traits (e.g. control, absence of cancer, absence of neurological disorders, etc.). Another pertinent challenge is the lack of universal standardization of variant interpretation, with data pointing towards high variability between computational algorithms and an inherent bias towards well-studied genetic diseases – hence, dependence on phenotype.^6^ Ironically, our efforts to analyze big data for personalizing medicine may have resulted in the opposite, ie, generalizations associated with populations and groups.

History has shown that great clinical and scientific lessons can be learned from rare disorders. For example, germline *PTEN* mutations cause a subset of Cowden syndrome,^7^ but each component cancer belonging to this syndrome can be common in the general population or other differential diagnoses.^8^ Importantly, somatic *PTEN* mutations are one of the most frequent mutations across many sporadic malignancies.^9^ The discovery of *PTEN* as the Cowden susceptibility gene emanated from the interrogation of a focused set of five meticulously-phenotyped families having individuals with full-blown disease.^7,10^ Therefore, for rare Mendelian disorders, it is only pragmatic to focus on deeply-phenotyped individuals to obtain the critical data that enables the practice of evidence-based, precision healthcare. Other studies have further emphasized the importance of “smart” experimental design, starting from a well-selected group of patients perfectly matched to controls to derive clinically-relevant conclusions.^11^ It is this “smart” experimental design coupled with well-annotated phenotypes that has led to identifying *PRDM1* in the etiology of therapy-induced second malignancies after Hodgkin’s lymphoma. Though “smart” experimental design in the setting of deep phenotyping seems common sensical, the recent popular opinion is that power is always in the numbers.

Deep phenotyping not only encompasses objectively documenting disease manifestations, but also focuses on integrating these data for a more organismal view. Appropriately, the “human phenomic science” approach of integrating human phenotypic data with physiologic, multi-omic, and imaging data has emerged as a blueprint for precision medicine.^12^ Indeed, the notion to deeply phenotype a finite set of individuals with a particular phenotype lies at the opposite end of the spectrum relative to large-scale population genomics. From these deeply-phenotyped individuals, it is then possible to identify physiologically relevant measures of disease risk that may then be extrapolated into other individuals with the same underlying etiology. Certainly, deep and accurate phenotyping enables using a smaller subset of patients to derive clinically meaningful and translational insights on disease etiology. Such an approach is particularly powerful to account for potential modifiers of disease risk (e.g., microenvironment, microbiome, family history, longitudinal follow-up, etc.) as pertinent to a real-life scenario. In the cancer realm, studies have also highlighted “exceptional responders” to chemotherapy (often n-of-1 cases), whose multi-faceted data analyses resulted in redesigned clinical trials for individuals with the same disease.^13,14^ Undoubtedly, in certain contexts, individual-level research outputs may pose more stringent regulatory policies to safeguard the data, which may limit timely and equitable access if left poorly streamlined.^15–17^

While it is instinctive to find security in the expanse of data, it is perhaps wise to heed the words of the Royal Society motto: *nullius in verba* (‘take no man’s word for it’) to understand the quality, type, context-dependence, clinical utility and limitation of data generated to investigate the human condition. At the core of all such efforts lies the patient. The present excitement and investment in precision medicine stems, in part, from the tremendous progress in how patient registries and databases have allowed us to understand hereditary syndromes and paved the way for both gene-directed therapies and preventative medicine. One challenge with data accruing rapidly is ensuring the integrity and quality control of data collection and curation, especially that of objectively documented phenotypic data. To obtain the data to enable evidence-based practice of precision healthcare, it may be necessary to wisely integrate large-scale population data with deep phenotyping data. An equally paramount issue will be educating researchers and clinicians alike, particularly due to increased reliance on such data to reflect “truths” especially with our desire to accelerate the translation of such data into clinical practice.

It is not in numbers, but in unity, that our great strength lies; yet our present numbers are sufficient to repel the force of all the world.

– Thomas Paine, Common Sense
